# A randomised crossover trial of nitrate and breakfast on prefrontal cognitive and haemodynamic response functions

**DOI:** 10.1038/s41538-024-00308-4

**Published:** 2024-09-13

**Authors:** Emerald G. Heiland, Frida Lindh, Callum Regan, Örjan Ekblom, Karin Kjellenberg, Filip J. Larsen, Maria Fernström, Gisela Nyberg, Maria M. Ekblom, Björg Helgadóttir

**Affiliations:** 1 Department of Physical Activity and Health, The Swedish School of Sport and Health Sciences (GIH), Stockholm, Sweden; 2 Department of Surgical Sciences, Medical Epidemiology, Uppsala University, Uppsala, Sweden; 3 Division of Physiotherapy, Department of Neurobiology, Care Sciences and Society, Karolinska Institutet, Huddinge, Sweden; 4 Division of Nursing, Department of Neurobiology, Care Sciences and Society, Karolinska Institutet, Huddinge, Sweden; 5 Department of Physiology, Nutrition and Biomechanics, The Swedish School of Sport and Health Sciences (GIH), Stockholm, Sweden; 6 Department of Global Public Health, Karolinska Institutet, Solna, Sweden; 7 Division of Insurance Medicine, Department of Clinical Neuroscience, Karolinska Institutet, Solna, Sweden

**Keywords:** Neurophysiology, Infrared spectroscopy

## Abstract

It remains unknown whether dietary nitrate and breakfast may enhance working memory (WM) performance by augmenting physiological mechanisms and subjective psychological well-being. We performed a 3-arm randomised within-subject crossover study, with pretest-posttest comparisons, to test whether nitrate consumption via breakfast with a beetroot juice shot or regular breakfast compared to no breakfast improved WM (measured with n-back tests) and cognitive task-related changes in prefrontal cortical haemodynamic response (oxygenated- and deoxygenated-haemoglobin derived from functional near-infrared spectroscopy). In addition, effects on peripheral vascular function and self-reported psychological factors were assessed. In 60 adolescents (13–15 years old; 66% girls), WM improved in all conditions, with no intervention effects. Intervention effects were seen for oxygenated-haemoglobin changes, such that it increased after the breakfast with a nitrate shot during the WM tests and decreased after the regular breakfast. Thus, different neurophysiological mechanisms may be at play to preserve WM in adolescents depending on their breakfast composition. The trial was registered in the ISRCTN registry (ISRCTN16596056) on 21/02/2022.

## Introduction

Nitrate rich foods such as green leafy vegetables and beetroot have been found to acutely improve working memory performance in older adults^1^, however neither of these effects nor the underlying mechanisms have been investigated in adolescents. Working memory is an important component of the cognitive domain of executive function, which involves memorisation and manipulation of information mentally over a short period of time. This prefrontal-cortex dependent function has been shown to be important for scholastic success^2^. However, previous studies in adults have produced conflicting results due to small sample sizes and methodological inconsistencies^3^. Well-powered studies in adolescents are needed to further disentangle these effects.

Under some conditions, high nitrate ingestion has been shown to induce a physiological response^4^, involving increases in baseline cerebral blood flow, which may in turn lead to improvements in cognitive performance at rest^5,6^. This potential improvement in cognitive performance from the nitrate-nitrite-nitric oxide (NO) pathway may enhance neurovascular coupling^7–9^. One study found in older adults that a high intake of nitrate-rich foods (500 ml beetroot juice) increased regional cerebral perfusion in the prefrontal cortex^5^. In one acute randomized crossover study of young healthy adults (mean age 21.3 years), intake of dietary nitrate from beetroot juice (450 ml = ~5.5 mmol nitrate) improved working memory on a revised serial subtraction task compared to the placebo condition^1^. In addition, they found that cognitive task-related changes in cortical haemodynamic response function partly explained the effects on cognition. Cortical haemodynamic response function was measured simultaneously with the cognitive test using functional near-infrared spectroscopy (fNIRS)^1^. Yet, it remains unknown if similar effects would occur in adolescents.

Not only specific dietary components, but also breakfast itself may be an important meal for cognitive performance. Many previous studies in children and adolescents have examined the immediate effects of breakfast on different cognitive domains, but with mixed results^10^. Neither the effects of having breakfast on working memory nor changes in the haemodynamic response as a potential mechanism, have been well tested in any age group. One study investigated breakfast consumption on the blood-oxygen-level-dependent (BOLD) signal from functional magnetic resonance imaging (fMRI), showing higher cognitive task-related activation after breakfast compared to fasting in adolescents^11^. Another study of female university students found that skipping breakfast compared to having breakfast resulted in a lower number of correct answers on an arithmetic test, along with lower levels of fNIRS-measured deoxygenated haemoglobin, but higher levels of a tissue oxygenation index^12^. Higher brain activity measured with fNIRS is often marked by an increase in oxygenated haemoglobin and a decrease in deoxygenated haemoglobin. However, in this study there was no difference in changes in concentration of oxygenated haemoglobin between eating or skipping breakfast, suggesting that oxygenated haemoglobin was not adequately used when breakfast was omitted, and that breakfast does affect cerebral activation. Despite the numerous conveyed health benefits from breakfast consumption in adolescents^10^, observational research has reported the prevalence of breakfast skipping among adolescents 12–17 years old to be 29% across nine European countries (*n* = 2929)^13^, and females to more likely skip breakfast than males^13^. A Swedish study estimated around 40% of Swedish adolescents to skip breakfast at least once per week^14^. More robust studies are needed to identify the pathways by which breakfast may sustain cognitive function in younger populations to develop more rigorous recommendations regarding breakfast in schoolchildren.

Other vascular effects are also reported to occur from intake of high amounts of nitrate and from breakfast, such as reduced blood pressure and arterial stiffness^6,15–18^, by increasing the bioavailability of NO through vessel dilation. Furthermore, breakfast consumption compared to skipping breakfast has also been found to counter undesirable psychological perceptions, such as negative mood and tiredness in adolescents^19,20^. In turn, improved feelings of well-being may boost motivation and attention to improve performance on challenging cognitive tasks^10^.

Therefore, the primary aim of this study was to assess the effects of having a high-nitrate breakfast (regular breakfast supplemented with nitrate provided through concentrated beetroot juice) on working memory using n-back tests in adolescents compared with a regular breakfast (referred to as low-nitrate breakfast) or no breakfast. Secondly, we aimed to test potential underlying mechanisms, such as changes in cognitive task-related haemodynamic response in the prefrontal cortex, arterial stiffness, and psychological factors, from having a high-nitrate breakfast compared with a low-nitrate breakfast or no breakfast. Thirdly, we aimed to examine the effects of having a regular breakfast (defined as low-nitrate breakfast) compared with no breakfast on working memory, cognitive-task-related haemodynamic response, arterial stiffness, and psychological factors. Finally, we tested whether salivary nitrite levels (as an indicator of nitrate) differed across breakfast conditions. This is the first study to investigate these questions in adolescents.

Based on the aforementioned research showing increases in cortical activity from dietary nitrate, we hypothesised that dietary nitrate would increase cortical haemodynamic response thereby improving working memory performance compared to having no breakfast or a low-nitrate breakfast. In addition, as skipping breakfast has previously been shown to be detrimental for cognitive performance, we hypothesised that having breakfast compared to breakfast omission would augment working memory performance via increased cortical haemodynamic response and improve peripheral vascular function and subjective psychological well-being.

## Results

There were 60 adolescents that participated in the study, of which 20 were classified by the investigator as boys and 40 as girls. Due to technical issues or participants not completing measurements, some data were missing in some conditions (see Supplementary Table 2). On average participants were 14 years old (standard deviation 0.9; range: 13–15 years), with an average body mass index of 20.1 kg/m^2^ (standard deviation 3.0), and 40 participants (66.7%) reported eating breakfast every weekday. The median kilocalories (kcal) of dinner the night before the no breakfast condition was 501 kcal (interquartile range: 370–645), on the regular breakfast condition was 454 kcal (401–670), and the night before the high-nitrate condition was 544 kcal (378–737). Deviations in median kilocalories for dinner before test days between conditions ranged from −191 to 127 kcal.

Average nitrite values were similar across all time points in the no breakfast condition (time point 0 = 11.8, time point 1 = 12.3, and time point 2 = 12.8 pg/ml). In the low-nitrate (regular) breakfast condition average nitrite levels increased slightly (to 13.4, 51.3, and 56.5 pg/ml), and larger increases were seen in the high-nitrate breakfast condition (to 13.8, 115.3, and 162.1 pg/ml). The median kcal of the regular breakfast was 496 and the high-nitrate breakfast was 493. Details regarding macronutrients at the two breakfast conditions can be found in Supplementary Fig. 4.

### Working memory

**Fig. 1:**
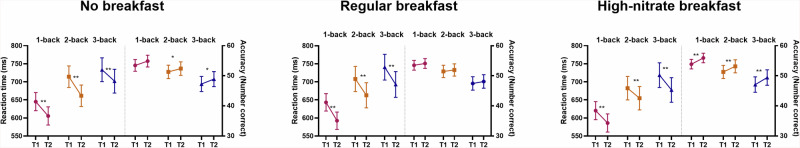
Within condition changes in working memory performance. Changes in reaction time and accuracy in the experimental conditions for the 1-, 2-, and 3-back tests from time point 1 (T1) to time point 2 (T2). *P < 0.05; **P < 0.01. (*N* = 60).

There were statistically significant improvements in reaction time on the 1-, 2-, and 3-back tests for all the conditions (Fig. 1; Supplementary Table 3). Accuracy statistically significantly improved only for the 2- and 3-back tests during the no breakfast condition (2-back β1.1(95% CI 0.1–2.1); 3-back: 1.6 (0.4–2.9)), with no significant changes observed in the regular breakfast condition for any n-back test. In the high-nitrate condition, improvements were seen in accuracy for all three n-back tests (1-back: 2.1 (0.8–3.3); 2-back: 1.9 (0.7–3.1); 3-back: 2.3 (1.1–3.6)). These improvements were not statistically significantly different between the conditions. In the stratified analysis by sex (Supplementary Fig. 5), the results were similar between boys and girls.

### Cortical haemodynamic response

**Fig. 2:**
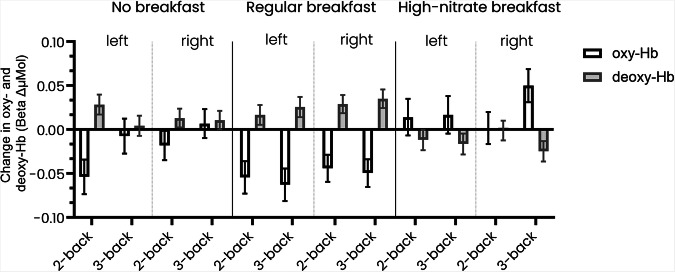
Within condition changes in oxygenated and deoxygenated haemoglobin concentrations. Change from time point 1 to time point 2 of oxygenated (oxy-Hb) and deoxygenated (deoxy-Hb) haemoglobin in the 2-back and 3-back tests relative to the 1-back for the different experimental conditions (*N* = 60), stratified by left and right prefrontal cortex hemispheres.

There were significant decreases in oxy-Hb and increases in deoxy-Hb in the left hemisphere during the 2-back during the no breakfast condition (oxy-Hb: β-0.054 (95% CI −0.074–−0.034); deoxy-Hb 0.028 (0.017−0.040)) (Fig. 2; Supplementary Table 4), and also during the regular breakfast in both the 2-back and 3-back in both the right and left prefrontal cortex. In the high-nitrate breakfast there were significant increases in oxy-Hb in the right prefrontal cortex during the 3-back (0.050 (0.031–0.069)) and decreases in deoxy-Hb in the left and right prefrontal cortex (left: −0.017 (−0.028–−0.005); right: −0.025 (−0.036–−0.013)). Cortical haemodynamic changes by sex can be found in Supplementary Fig. 6.

**Fig. 3:**
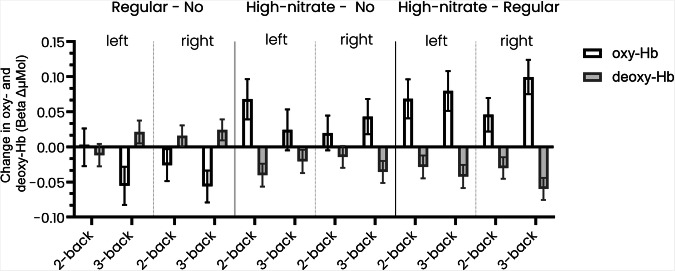
Between condition effects for changes in oxygenated and deoxygenated haemoglobin concentrations. Intervention effects for change from time point 1 to time point 2 and between condition of oxygenated (oxy-Hb) and deoxygenated (deoxy-Hb) haemoglobin in the 2-back and 3-back tests relative to the 1-back for the different experimental conditions (*N* = 60), stratified by left and right prefrontal cortex hemisphere.

Intervention effects were seen between the breakfast conditions (Fig. 3; Supplementary Table 5). Effect sizes can be found in Supplementary Table 5. There were significant decreases in regular breakfast compared to the no breakfast condition in the 3-back in oxy-Hb but increases in deoxy-Hb. Comparing the no breakfast to the high-nitrate breakfast there were significant increases in oxy-Hb in the 2-back and the 3-back tests but decreases in deoxy-Hb. Comparing the high-nitrate to the regular breakfast there were significant increases in oxy-Hb and decreases in deoxy-Hb in both the 2-back and 3-back.

In the sensitivity analyses, some differences could be observed across different anatomical landmarks of the prefrontal cortex (Supplementary Tables 6 and 7). In the high-nitrate breakfast compared to no breakfast, there were significant intervention effects in oxy-Hb for the 3-back in the right frontopolar area (BA 10; 0.079 (0.043–0.115)) and right dorsolateral prefrontal cortex (BA 46; 0.015 (0.003–0.028)).

### Psychological factors

**Fig. 4:**
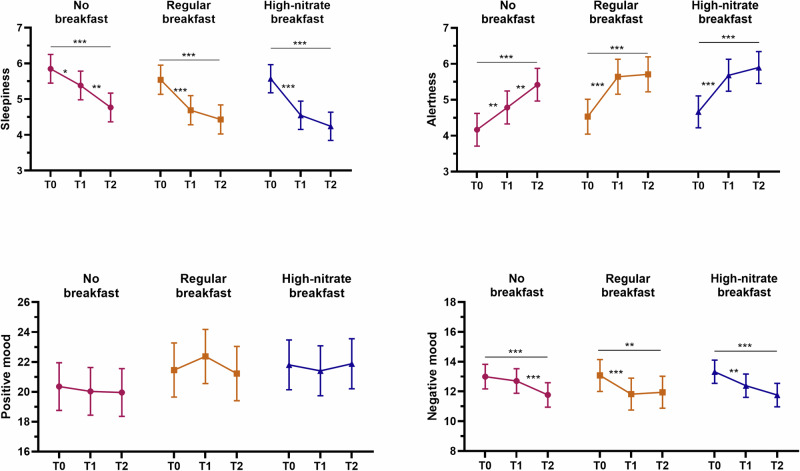
Within person changes in psychological factors across conditions. Within person changes from time points 0, to 1, to 2 in sleepiness, measured using the Karolinska Sleepiness Scale (higher score indicates more awake); alertness assessed using a 10 cm visual analogue scale (with higher score indicating more alert); positive and negative mood derived from the PANAS with higher scores indicating stronger positive/negative feelings, across the experimental conditions (*N* = 60). *P < 0.05; **P < 0.01; ***P < 0.001.

The results from the psychological measures can be found in Fig. 4 and Supplementary Table 8. The KSS showed decreased sleepiness and the VAS showed increased alertness across all conditions between time point 0 and time point 1. During the no breakfast condition there was a significant decrease in sleepiness and an increase in alertness between time points 1 and 2 but not for the regular and high-nitrate breakfasts. The PANAS positive did not change over time in any conditions, but the PANAS negative decreased between time point 0 and both time points 1 and 2 for the regular and high-nitrate breakfasts. In the no breakfast condition, there was a significant decrease between time points 0 and 2 as well as time points 1 and 2. No significant intervention effects were found for the psychological factors.

### Peripheral vascular measures

**Fig. 5:**
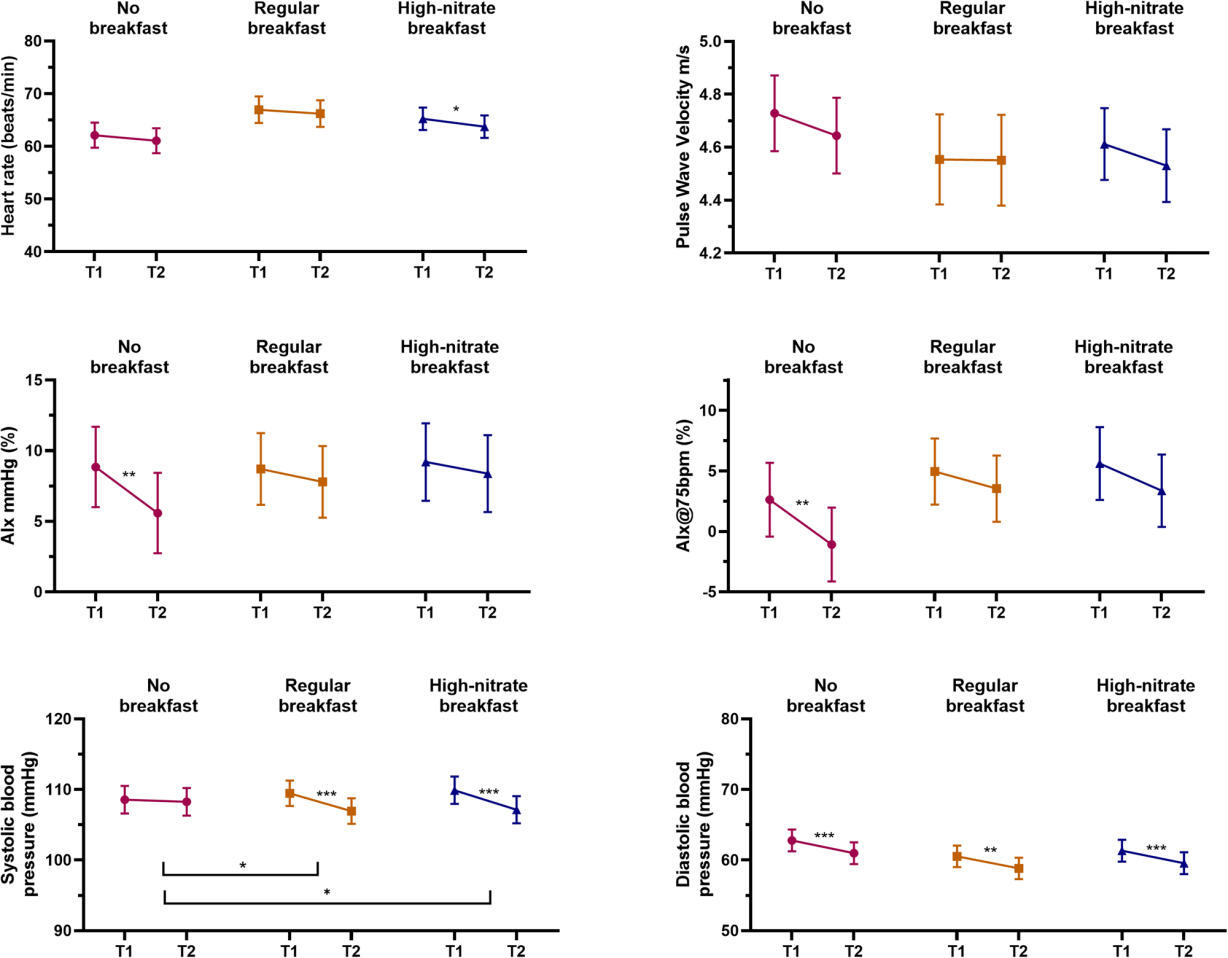
Within person changes in arterial stiffness measures, heart rate, and blood pressure. Within person changes from time point 1 to time point 2 for pulse wave velocity (m/s), augmentation index (%), augmentation index 75 (%), systolic and diastolic blood pressure (mmHg), and heart rate (beats per minute) in the different experimental conditions (*N* = 60). *P < 0.05; **P < 0.01; ***P < 0.001.

Figure 5 and Supplementary Table 8 show the results from the arterial stiffness measures as well as for heart rate and blood pressure (diastolic and systolic). AIx and AIx@75 only significantly decreased in the no breakfast condition. There were no significant changes in PWV. No significant intervention effects were seen for AIx, AIx@75, or PWV. Heart rate did not significantly change for the no breakfast and regular breakfast conditions but decreased in the high-nitrate breakfast, although no significant intervention effects were found. No significant changes were observed for the no breakfast condition for systolic blood pressure, while significant decreases were seen for both breakfast conditions. There was a significant interaction effect, as the changes in the high-nitrate breakfast condition were significantly different from the changes in the no breakfast condition. There was also a significant intervention effect between the no breakfast and the regular breakfast condition. Diastolic blood pressure decreased across all conditions with no significant intervention effects.

## Discussion

This is the first study to assess the acute effects of dietary nitrate on cognitive performance in adolescents and the potential underlying mechanisms. The results from our study showed an improvement in both accuracy and reaction time of the working memory tests in all three conditions (no breakfast, low-nitrate (regular) breakfast, and high-nitrate breakfast), except that reaction time did not improve in the low-nitrate breakfast condition. The changes in accuracy and reaction time however were not different between the conditions although effects in accuracy were more consistently seen in the high-nitrate breakfast condition. Despite there being no statistically significant differences on cognitive performance between the conditions, the cerebrovascular responses, coupled with the improvements in working memory performance, did differ between the conditions. Oxy-Hb decreased and deoxy-Hb increased during the 2-back and 3-back tests in the regular breakfast (low-nitrate) condition, whereas during the high-nitrate condition oxy-Hb increased and deoxy-Hb decreased, particularly for the 3-back task.

Regarding the other outcomes, among the psychological factors, only mood improved after the no breakfast condition, but it was not different from the breakfast conditions. Similarly, no intervention effects were seen for any of the arterial stiffness measures. Moreover, systolic blood pressure significantly decreased after both breakfast conditions, with the change being significantly different from the no breakfast condition. In addition, heart rate decreased only after the high-nitrate breakfast, but this was not significantly different from the other two conditions. Therefore, some cerebrovascular and peripheral vascular changes may be at play from nitrate and breakfast consumption in adolescents, influencing task performance.

Previous research has shown mixed results for the effects of nitrate on cognitive performance and cerebrovascular responses^21^. However, all these previous studies have been performed in adult populations and most involved prolonged supplementation. Therefore, there is a lack of studies looking at acute effects and focusing on adolescent populations.

Five previous clinical trials have investigated the acute effects of nitrate on cognitive performance in adults^21^. Despite the consistency of beetroot juice being the main source of dietary nitrate supplementation in these earlier studies, they have differed in dosage and the cognitive tests employed. Duration from time of ingestion has been relatively consistent, being between 90 and 120 min, and populations investigated have been in healthy, young (average age between 20 and 24 years) adults, with sample sizes ranging between 10 and 40 participants. The results of these studies have mainly demonstrated no acute effects of nitrate ingestion on cognitive performance. However, it is challenging to make strong conclusions due to the diversity of cognitive tests utilised between these studies. Indeed, Wightman et al. observed improvements on cognitive performance among those who received beetroot juice compared to those who received the placebo, specifically in the serial 3 subtraction test, which captures accuracy^1^. The other cognitive test employed in Wightman et al.’s study measured processing speed (Rapid Visual Information Processing test (RVIP)) with no improvements observed after ingesting beetroot juice^1^. However, they note that cognitive task performance in the treatment group was much lower at the baseline compared to the placebo group. Results from Wightman’s study may have also been tainted by a small sample size; yet, it has been the largest reported study prior to this current study. Another study also saw positive effects of beetroot juice on a prefrontal cortex-dominant task in 36 male team-sport players^22^. The study’s results demonstrated that ingestion of beetroot juice improved reaction time in the Stroop test, but not in accuracy. On the other hand, participants ingested nitrate over 5 days. Similarly, Gilchrist et al. also saw improvements on reaction time in patients of type 2 diabetes who supplemented with nitrate over 2 weeks^23^. However, these studies have had longer periods of supplementation compared to the current study, whereas the immediate effects need to be studied further as they can provide important information on the day-to-day impact on an adolescent’s performance at school. In the current study, improvements were seen in reaction time but not accuracy in the low-nitrate condition, whereas in the high-nitrate condition improvements were seen on both reaction time and accuracy. However, no differences in cognitive performance were observed between the conditions. Thus, even with a larger sample size and improvements in working memory seen within conditions, there were no differences between conditions, which are in line with most previous studies in adults. More studies are needed in adolescents to confirm this result using more comparable cognitive tests.

Concerning the mechanisms, increased NO availability through ingestion of nitrate-rich food such as beetroot is postulated to have physiological responses that underly potential improvements in cognitive function. In the brain, NO has many important functions, such as neurotransmission, vasodilation, and neurovascular coupling^8,9^. Wightman et al. also measured fNIRS derived prefrontal cortical haemodynamic changes^1^. This study found beneficial effects of nitrate on the haemodynamic response with corresponding improvements seen in the serial 3 subtraction cognitive test compared to the placebo. However, effects were only observed for total haemoglobin and not for deoxy-Hb; effects for oxy-Hb were not reported. Furthermore, a reduction in total haemoglobin during the RVIP test was found. The results of the current study exhibited no intervention effects for cognitive performance, but changes in oxy- and deoxy-Hb were evident and differed by nitrate condition, as hypothesised. Increases in oxy-Hb were observed, in the present study, during the 3-back of the high-nitrate condition and a decrease of oxy-Hb during the low-nitrate breakfast condition. Therefore, different mechanisms may be at play in the different breakfast conditions. The high-nitrate consumption may have modulated the haemodynamic response to perform well on both tasks of accuracy and reaction time. In the high-nitrate breakfast the vasodilator and enhancing neurovascular coupling effects of NO, as seen in an fMRI study^7^, could explain the changes observed in the haemodynamic response. Indeed, a study looking at cerebral perfusion using arterial spin labelling found that dietary nitrate improved regional brain perfusion in older adults, specifically in the frontal lobe where tasks of executive function and working memory primarily occur^5^. This also indicates that even within the prefrontal cortex, haemodynamic responses from nitrate may differ by brain region. In our sensitivity analyses we observed increases in oxy-Hb during the 3-back in the high-nitrate breakfast in the left pars triangularis, middle frontopolar area, and right dorsolateral prefrontal cortex (Supplementary Table 6), among some of which these increases were significantly different between breakfast conditions (Supplementary Table 7). Although fNIRS does not allow for study of deeper brain regions and previous studies have not delved further into brain parcellations particularly in regard to nitrate or breakfast effects on hemodynamic response, future region-specific analyses can provide more information on localized responses to interventions. During the low-nitrate condition, decreases occurred in oxy-Hb in the present study, which may relate to what was observed in Wightman’s study during the RVIP^1^. On the other hand, the decrease in oxy-Hb with improvement of working memory function could also be explained as being a neural efficiency effect where less oxy-Hb was needed to perform well in reaction time.

The effects of NO from the beetroot juice are also evident in the decrease in systolic blood pressure and heart rate present in the high-nitrate condition in the present study (Fig. 5; Supplementary Table 8). Nitrate consumption has been proposed to be efficacious in reducing blood pressure^6,17^ by decreasing peripheral vascular resistance^16^. One study of 12 young-adult females found that those who ingested beetroot juice compared to controls had a reduced cerebrovascular resistance, systolic blood pressure, and total vascular resistance. However, they also observed that middle cerebral artery velocity was significantly higher when beetroot juice was consumed compared to the control drink of orange juice^6^. In the present study, despite the decrease in systolic blood pressure for the high-nitrate condition, we saw no changes in peripheral arterial stiffness. This may be due to our sample being young and healthy.

Surprisingly, no improvements were seen on self-assessed psychological factors of sleepiness, mood, or alertness from nitrate ingestion compared to the low-nitrate (regular) breakfast, or no breakfast in the present study. Previous nitrate studies have not taken these factors into account; however, breakfast studies have. Earlier studies testing breakfast consumption versus omission have indicated improvements on subjective feelings from breakfast compared to fasting^10,19,20,24^, which were proposed to affect cognitive performance indirectly^10^. Contrarily, in the current study we did not see differences in psychological factors by breakfast consumption, which may also explain the lack of differences seen in working memory test performance.

When comparing breakfast (low-nitrate (regular) breakfast) to no breakfast, there were no significant differences in working memory. However, in the regular breakfast condition reaction time increased but no change occurred in accuracy. A systematic review by Adolphus et al. reported mixed results of the acute effects of breakfast versus breakfast omission on different cognitive tests in children and adolescents^10^. They found that in the 13 studies that examined tests of executive function (including working memory) a positive effect of breakfast compared to skipping breakfast was seen in seven of these studies^10^. In one of the studies executive function was enhanced under fasting conditions compared to consuming breakfast, however these improvements were specific to well-nourished children. This may explain the lack of difference in the no breakfast to breakfast condition in our study, as most participants were healthy and well-nourished.

Moreover, although breakfast consumption overall has exhibited beneficial effects on cognitive function in previous studies in adolescents and children, some variations were observed by sex^10^. In contrast, we did not see an interaction effect of sex and time on changes in cognitive performance (Supplementary Fig. 5). In studies specifically examining the effects of breakfast compared to no breakfast on working memory in adolescents, mixed results have been observed^20,25^. Adolphus et al. also concluded in their review that breakfast with a low glycemic index was consistently associated with positive effects on attention^10^. Some possible reasons for this effect of breakfast on cognition is that increased blood glucose may improve cognition by facilitating neuronal glucose uptake in the brain regions where extracellular glucose concentrations are decreased during times of high neuronal glucose uptake^10^. In addition, changes in concentrations of neurotransmitters and hormones, may mediate the changes to cognition, while also glucose ingestion may increase acetylcholine synthesis, which could influence cognitive function^10^. Specifically, cortisol secretion because of the combination of carbohydrate consumption and an arousing situation (cognitive testing) may interact to facilitate effects on cognitive performance. Ingestion of glucose can interact with a stressful task and provoke a greater cortisol response^10^. This suggests that the postprandial blood glucose profile may mediate the effects of breakfast on cognitive performance. Although we did not collect blood samples in the present study to test blood glucose and confirm this theory, we saw no difference in working memory performance between the breakfast condition which had a low glycemic index and the no breakfast condition. Therefore, blood glucose changes may not explain the results seen in our study, but further research is needed to disentangle the effects of glucose on cognitive performance.

Furthermore, it has been suggested that tasks with higher cognitive demands may be more sensitive to nutritional manipulations^19,20^. One study found that the response on a high load working memory test improved 120 min post consumption of a low glycemic index breakfast compared to breakfast omission^20^. It is possible that the n-back tests that were employed in the current study were not difficult enough to see this effect.

Studies examining mechanisms related to cognitive performance from breakfast have been few. One study observed that skipping breakfast had negative effects on arithmetic test performance with reduced deoxy-Hb changes and no changes in oxy-Hb, measured with fNIRS, in healthy female university students^12^. Indeed, we did see a difference in the haemodynamic response between the no breakfast and the regular breakfast condition, with significant differences in the 2-back and 3-back in oxy-Hb in the prefrontal cortex. Thus, having breakfast influenced the cerebrovasculature. A study by Fulford et al. tested 21 adolescents’ brain activity using fMRI during n-back tasks in satiated and fasted conditions^11^. It was found that participants were faster on the 1-back and 2-back tests after breakfast compared to the no breakfast, however, the differences between the conditions were not significantly different from each other. Similar to our study, Fulford et al. also saw an increase in activation from breakfast relative to the fasted state for both n-back tests although with no improvement on n-back test performance^11^. The increased activation in Fulford et al.’s study indicates greater attentiveness, and the areas activated were associated with the demands of the n-back test (Brodmann area 45 in the frontal cortex). There may have been greater efficiency when performing the cognitive test after having breakfast than no breakfast on reaction time. Greater activity has been reported in the prefrontal cortex when conducting n-back tasks, after a nutritionally balanced breakfast compared to no breakfast^26,27^. However, studies investigating the underlying mechanisms are lacking and whether changes in the haemodynamic response may explain the differences in between breakfast and no breakfast is still to be researched. Indeed, understanding these mechanisms is crucial, especially considering the long-term implications of skipping breakfast on cognitive and academic performance. However, it is essential to note that the present study focused solely on acute effects. In children, cognitive function may be more susceptible to short-term modifications of diet due to their high metabolic rates and higher energy demands^28^. Previous studies have indicated immediate beneficial effects of eating breakfast in children, such as improved memory, attention, and visual discrimination^28^. Therefore, it is important to acquire a greater understanding of the acute effects to develop interventions that aim to improve cognitive performance in schoolchildren.

One limitation was that the food components provided at breakfast may have hindered the absorption of nitrate. However, part of the aim of the study was to examine a breakfast composition that included higher levels of nitrate to be more aligned with everyday meal preparation for schoolchildren. Participants were also provided with breakfast options that had low nitrate content and most ate breakfasts of similar compositions and energy levels across the two breakfast conditions. Therefore, the effects reflect mainly the intervention of the beetroot juice supplement. Deviations could have occurred in kcal consumed within-person that may have altered the results, but the deviations were minimal between conditions. This study involved healthy adolescents from an urban area; thus, careful consideration needs to be made when generalising to other populations. Furthermore, as blood samples were not taken in this study, the optimal point where nitrate peaks could not be individually determined. Previous literature, allowed us, on the other hand, to estimate what is considered the time window of when this would occur. In addition, the lack of pseudo-randomisation of the n-back tests may have led to increased anticipation, yet, alternatively, confusion was minimised to produce more reliable results. The haemodynamic response was only measured in the prefrontal cortex in the current study. A broader understanding of the activation could have been retrieved with a cap covering all regions of the cortex. However, the focus was on working memory, which is highly associated to the prefrontal cortex, and we estimated sub-regions of this cortical area. The lack of additional measurements during the fNIRS recordings reduced chances of removing other potential physiological confounders that are highly correlated with the fNIRS signals, such as heart rate and blood pressure. Short-separation channels were included, which were used to remove the most problematic physiological confounders in the cortical and extracerebral layers. Comparisons with fNIRS studies lacking methodology to extract superficial blood flow should be performed with caution. This study is also one of the largest to date to study breakfast and cognitive performance in adolescents and the first to study dietary nitrate supplementation and working memory in adolescents. The randomised crossover design also contributed to increasing the robustness of the results, as the participants were used as their own controls, thus reducing any confounding bias. Furthermore, this study exposed participants to minimal harm. The lack of blinding could introduce bias due to anticipation, however, the randomization of conditions helped to minimize this bias.

In summary, supplementation of nitrate or having breakfast compared to no breakfast or nitrate showed no difference in working memory performance in adolescents. However, the cerebrovascular mechanisms differed by breakfast composition. Dietary nitrate increased oxy-Hb and reduced deoxy-Hb, compared to both the no and regular breakfast conditions. When comparing regular breakfast to no breakfast, oxy-Hb decreased and increased deoxy-Hb. Therefore, nitrate may induce beneficial cerebrovascular effects, whereas having breakfast may lead to neural efficiency compared to not having breakfast. Further studies are needed to verify these results and for replication, nonetheless this study sheds light on the potential health effects of nitrate and breakfast, which may be important for school performance in adolescents.

## Methods

### Study design and participants

A detailed description of the protocol and a priori hypotheses can be found elsewhere^29^. We performed a 3-arm randomised within-subject crossover trial with pretest-posttest comparisons. Participants were recruited among pupils in grades 7–9 from schools in the Stockholm region who could understand Swedish. Those who were diagnosed with diabetes, epilepsy, vascular health conditions/circulatory abnormalities, or had visual/auditory impairments were excluded. Familiarisation visits occurred at the local schools or in a laboratory at the Swedish School of Sport and Health Sciences (GIH) in Stockholm, Sweden. Recruitment was from February 25, 2022 to November 8, 2022. Recruitment ended after a sufficient number of students were recruited.

The three experimental conditions were randomised and occurred on three separate days, taking place at the laboratory, with a minimum washout period of six days to reduce any carry-over effects^30,31^. The 24-h before each experimental day, monitoring and standardisation of physical activity, sleep, and dietary intake were performed. Participants received a 500 SEK (~€47) gift card for their participation.

### Ethical approval

Ethical permission was received from the Swedish Ethical Review Authority, Stockholm, Sweden (Dnr: 2021-07053-01) according to the Declaration of Helsinki, and this trial was prospectively registered in the ISRCTN registry 10.1186/ISRCTN16596056 (ISRCTN16596056) on 21/02/2022. Written informed consent was obtained by each participant and their guardians prior to data collection.

### Familiarisation visit

During the familiarisation session, participants were introduced to the experimental day procedures and tests, such as the cognitive test, the fNIRS cap, the arterial stiffness measure, and the questionnaires. Other information collected included age, sex, head measurements (i.e., circumference, nasion to inion, and between pre-auricular points for fNIRS cap size and head registration in the analysis), food allergies or any dietary intolerances, and a health declaration questionnaire. Participants also filled in a short questionnaire regarding their frequency of consuming nitrate-rich foods (pictures of foods (e.g., spinach, beetroot) were provided) and their regular breakfast consumption. Self-reported information on scheduled physical activity was also recorded to help with maintaining consistency on physical activities scheduled on pre-test days.

Participants were paired to attend on the same day (with a 20 min gap in starting time) according to head size (for practicality in measuring fNIRS). Afterwards, a simple randomisation was performed, using a password-protected computer-generated random order method in Microsoft Excel. Paired participants were randomly assigned to a specific experimental condition order, only seen by the assigned researcher. Blinding of the researcher was not possible in this study design. The randomisation was not revealed to the participants until recruitment and familiarisation were completed.

### Pre-experimental monitoring and standardisation

Participants were asked to maintain consistency in their physical activity behaviours each day before test days, and to record details about their physical activity, diet, and sleep in a standardised diary. Participants wore accelerometers to monitor their physical activity and sedentary time during pre-test days (hip-worn Actigraph GT3X+ or wGT3X-BT) and sleep at night (wrist-worn Actigraph GT3X+ or wGT3X-BT). Actigraph provided information on total time spent in physical activity and percentages of awake time spent in sedentary, light, and moderate-to-vigorous physical activity, according to the standardised Evenson (2008) cut-points^32^. At night, participants wore the Actigraph GT3X+ on their left wrist^33^. Using an algorithm for sleep-wake scoring in children^34^, time to sleep onset, sleep efficiency, number of times waking up after sleep onset, and total sleep time were retrieved. The diaries provided complementary information regarding wake-up time, going-to-bedtime, and sleep quality.

Dietary information was recorded in the diaries to standardise dietary intake on pre-test days. Dietist Net Pro http://www.kostdata.se/se/dietist-net/dietist-net-pro software was used to analyse the nutrient intake of pre-experimental day meals. Participants avoided foods that were rich in nitrate on days before the experiment and fasted after dinner until they arrived at the laboratory the next morning. Additionally, participants were also prohibited from using chewing gum or anti-bacterial mouthwash on experimental test days as these can inhibit oral nitrate metabolism^18,35^.

### Experimental conditions

The three experimental conditions were: (A) no breakfast; (B) low-nitrate breakfast (regular breakfast); and (C) high-nitrate breakfast (regular breakfast supplemented with nitrate provided through concentrated beetroot juice). On test days, material from pre-experimental monitoring were collected (e.g., physical activity and diet diary). Height was measured only on the first test-day, while weight was measured on the day of the high-nitrate breakfast condition because the amount of beetroot juice provided was individualised to body weight. The amount of nitrate was restricted to the daily acceptable limit of 3.7 mg per kg body weight per day^36^. Participants arrived at the laboratory by taxi arranged by research staff. A practice run of the working memory test was performed, then a saliva sample (to assess nitrate/nitrite levels) was taken, followed by filling in questionnaires on psychological factors (mood, alertness, and sleepiness) before breakfast was provided (Supplementary Fig. 1).

On the first breakfast experimental day (either condition B or C), participants chose the quantity and type of food that closely resembled their regular breakfast consumption from the foods provided. The amount was documented and replicated for the next breakfast condition. Foods provided had a low glycaemic index: wholemeal bread, cheese, ham, cucumber, yoghurt, and low sugar muesli. A standardised amount of orange juice was provided after consuming the beetroot juice, to aid in its ingestion, due to the potential unfavourable taste of the beetroot shot. Orange juice was also provided at the regular breakfast condition for consistency. Water intake was allowed ad libitum but documented and matched for each experimental condition.

After time point 0 and the first measurements, the participants sat for 130 min to allow for the plasma nitrite to peak^37^ before the last measurements were taken. Participants were allowed to read books or listen to audio books related to their schoolwork. They were not permitted the use of technological devices or to perform any physical activity. One social break involving a 2–3 min chat with a research staff member was provided on each test day, at the same time.

### Primary outcome: working memory

Computerised n-back tests (1-, 2-, and 3-back)^38^ developed in E-Prime 2.0 (Psychology Software Tools) were used to assess working memory reaction time (ms) and accuracy (number of correct responses). The n-back test has been shown to be a robust test of working memory in children^39^. These were averaged across 3 blocks of 20-digit sequences, with each n-back lasting 35 s. Working memory was tested twice (during time point 1 and time point 2; Supplementary Fig. 1). By pressing a key, participants indicated if the digit presented on the screen was the same digit that was presented one digit earlier (1-back), two digits earlier (2-back), or three digits earlier (3-back) (Supplementary Fig. 2). Each digit was presented for 1500 ms with an inter-stimuli interval of 500 ms. Therefore, participants had 2000 ms to indicate whether the digit was correct or not. A white dot appeared on the screen for 20 s before each n-back test that the participant had to stare at and count from 0.

### Secondary outcome: cortical haemodynamic response derived from fNIRS

Cognitive task-related changes in oxygenated (oxy-Hb) and deoxygenated (deoxy-Hb) haemoglobin were measured via continuous wave functional near-infrared spectroscopy (fNIRS) (portable NIRSport, 8-8 system, with short-separation channels, NIRx Medizintechnik GmbH, Berlin, Germany) simultaneously as the n-back tests. NIRStar 15.2 software was used to capture the raw data, with a sampling frequency at 7.81 Hz at wavelengths 760 nm and 850 nm. The fNIRS cap had 8 LED light sources and 15 detectors (silicon photodiode (SiPD)), of which 7 of the detectors were long-separation and 8 short-separation. The set-up of long-distance source-detector optode pairs made 20-channels over the prefrontal cortex, placed according to the standard 10–20 EEG system (Supplementary Fig. 3 and Supplementary Table 1). The distance between the sources and detectors was 3 cm for long-separation channels and 0.8 cm for short-separation channels.

Room lights were dimmed and the fNIRS cap was fitted with the chinstrap prior to data acquisition for optimal signal quality. To replicate the position of the fNIRS cap on the participant’s head, the central location of the Cz point was located and the distance between the nasion and the Fpz point were aligned. The system was calibrated prior to each cognitive test session. In the case of low signal quality, the faulty channels were corrected either by moving hair with a cotton swab or gel, or the cap was repositioned. The edge of the cap on the forehead was marked after calibration for replicability for the second time point. Headphones were worn to reduce distractions.

### Secondary outcome: psychological factors

Psychological factors were assessed at time point 0 before the experimental conditions and at time points 1 and 2 (Supplementary Fig. 1). Mood was assessed using the Positive and Negative Affect Scale (PANAS)^40^ questionnaire. PANAS includes 10 positive and 10 negative moods that were rated on a 5-point scale (very slightly/not at all; a little; moderately; quite a bit; extremely). Positive affects and negative affects were separately summed. Each ranged from 10 to 50 with higher scores indicating a higher positive mood or higher negative mood. Alertness was measured using a 10 cm visual analogue scale (VAS); ranging from “not at all” to “completely alert”^41^. Sleepiness was measured using the 9-point Karolinska Sleepiness Scale (KSS) questionnaire^42^. The scale ranged from “extremely alert” to “very sleepy, great effort to keep awake, fighting to sleep”; with higher scores indicating higher levels of sleepiness.

### Secondary outcome: peripheral vascular measures

Arterial stiffness was measured as pulse wave velocity (PWV) and augmentation index (AIx and AIx@75) at time points 1 and 2 (Supplementary Fig. 1) using SphygmoCor XCEL PWA/PWV system^43^. AIx was defined as the difference between the first and second systolic peak and was expressed as a percentage of the pulse pressure. AIx@75 is similar to AIx but corrected for a heart rate at 75 beats per minute. After 2 min of rest, PWA was estimated from a brachial arm cuff where blood pressure and waveforms were derived. This was followed by measuring PWV from three high fidelity pressure waveforms using a carotid tonometer with a right leg cuff. PWV was assessed between carotid and femoral arteries and was calculated by the formula: PWV (m/s) = distance between measurement location (m)/transit time (s)^44^. The average of the three recordings was used to determine PWV (m/s)^45^.

### Secondary outcome: salivary nitrite concentrations

Nitrate levels were indicated through nitrite concentrations collected via three saliva samples: one before the experimental conditions (time point 0) and at two time points (time points 1 and 2) after the experimental conditions (Supplementary Fig. 1). Cotton buds in test tubes were used to collect the saliva. These samples were centrifuged and stored at −80 °C, until the time of analysis (<6 months from collection). The OxiSelect™ In Vitro Nitric Oxide (Nitrite/Nitrate) Assay Kit (Fluorometric) from Cell Biolabs, Inc. (San Diego, CA, USA) was used to measure nitrite (NO_2_^−^) levels collected from the saliva samples.

### Sample size

Calculation of the sample size has previously been published in the protocol article^29^. Briefly, the estimated sample needed was calculated as 43, based on the effect sizes of working memory performance and psychological outcomes and a drop-out rate of 20%. Assuming a two-tailed test and using an alpha of 0.05, beta of 0.8, and correlation of 0.5, the differences in mean and standard deviations for an effect size of 0.63 for nitrate on working memory, giving a sample size of 22. While a sample size of 20–36 was estimated for an effect size of 0.48–0.67 for the effects of breakfast on psychological factors.

### Statistical methods

Linear mixed effect models were used to test within condition changes in working memory reaction time and accuracy, psychological factors, arterial stiffness, and nitrate/nitrite concentrations from time point 0 to 1 or time point 1 to time point 2, using subject as random effect. Statistically significant differences in the change from time point 1 to 2 and between condition, to determine intervention effects, was tested using an interaction between time and condition. Stata/SE 17.0 (StataCorp, LLC, Texas, USA) was used to analyse the data. Sensitivity analysis addressing whether changes differed by sex was performed through stratification. A *P*-value ≤ 0.05 was considered a statistically significant effect.

Changes (Δµmol) in oxy-Hb and deoxy-Hb were estimated using MATLAB-based software NIRS Brain AnalyzIR Toolbox (https://github.com/huppertt/nirs-toolbox^46^) simultaneously with the working memory tests. Oxy-Hb was the main fNIRS outcome as it has previously been reported to be the most sensitive indicator of neural activation^47^. Quality check of each signal was performed by visually assessing the signals and checking the relative coefficient of variation (CV%) for each channel. A CV% less than 15% indicated good signal quality^48^. Poor signal quality was managed in the statistical analysis. Raw voltage was converted to optical density and subsequently to haemoglobin concentrations based on the modified Beer–Lambert law^49^. Data were processed through minimal manipulation as suggested by Santosa et al.^46^. First level statistics involved estimating the fNIRS parameters during the 1-, 2-, and 3-back tasks (each 35 s duration) relative to the baseline (20 s rest). Brain activation was predicted using a general linear model (GLM) with a canonical design matrix, using an age-adjusted differential pathlength factor^50^ and an autoregressive pre-whitening approach with iteratively reweighted least squares (AR-IRLS) for each source-detector pair. The AR-IRLS approach was chosen because it reduces false-discovery rate^51^. Short-separation regressors were used to control errors caused by systemic physiology and motion artefacts. The GLM yielded regression coefficients (betas) for each channel in each n-back task and condition that was used in the second-level analyses (group-level analysis). This entailed the coefficients of Δoxy- and Δdeoxy-Hb, averaged across the left and right prefrontal cortex separately, for each n-back task and condition. A False Discovery Rate (FDR) correction using a Benjamini-Hochberg procedure was used to correct for multiple comparisons (*q-value* = FDR-adjusted *p* ≤ *0.05*). Linear mixed effect models were employed with Δoxy- and Δdeoxy-Hb as dependent variables, with condition and time as fixed effects and subject as a random effect to assess within and between condition differences in cortical haemodynamic response parameters during each n-back task. Additional, linear mixed models were performed to assess intervention effects including time and condition as interaction terms. Sex stratified analysis was also performed. Cohen’s d was calculated to estimate effect sizes for between condition effects in sensitivity analyses.

### Sensitivity analysis

To estimate probe location over anatomical landmarks of the prefrontal cortex, the fNIRS Optodes’ Location Decider (fOLD) Matlab toolbox^52^, was used to assign optodes to specific regions of interest (ROI) based on the 10–20 EEG system and the Brodmann atlas (BA)^53^ (Supplementary Fig. 3 and Supplementary Table 1). Specificity was set at 30%, as recommended by Zimeo Morais et al.^52^, and symmetry was forced. With this approach our probe covered the following regions: pars triangularis Broca’s areas (BA 45, left and right), dorsolateral prefrontal cortex (BA 46, left and right; BA 9 left, middle, and right), orbitofrontal area (BA 11 left), and the frontopolar area (BA 10, left, middle, and right), which were then statistically analysed using same procedures as in the forementioned fNIRS analysis.

## Supplementary information

Supplementary Information

## Data Availability

Data will be made available only upon reasonable request by contacting: https://www.gih.se/forskning/forskningsgrupper-och-projekt/fysisk-aktivitet-hallbarhet-och-hjarnhalsa---e-pabs/om-forskningsgruppen. No customized computer code or algorithms were developed in this project.
