# Supplementary material for: A randomised crossover trial of nitrate and breakfast on prefrontal cognitive and haemodynamic response functions
Source: NPJ Sci Food. 2024 Sep 13;8:64. doi: 10.1038/s41538-024-00308-4 (PMC11399140; doi:10.1038/s41538-024-00308-4)
Supplement: Supplementary file 1 — Supplementary Information [file 41538_2024_308_MOESM1_ESM.pdf]

SUPPLEMENTARY INFORMATION

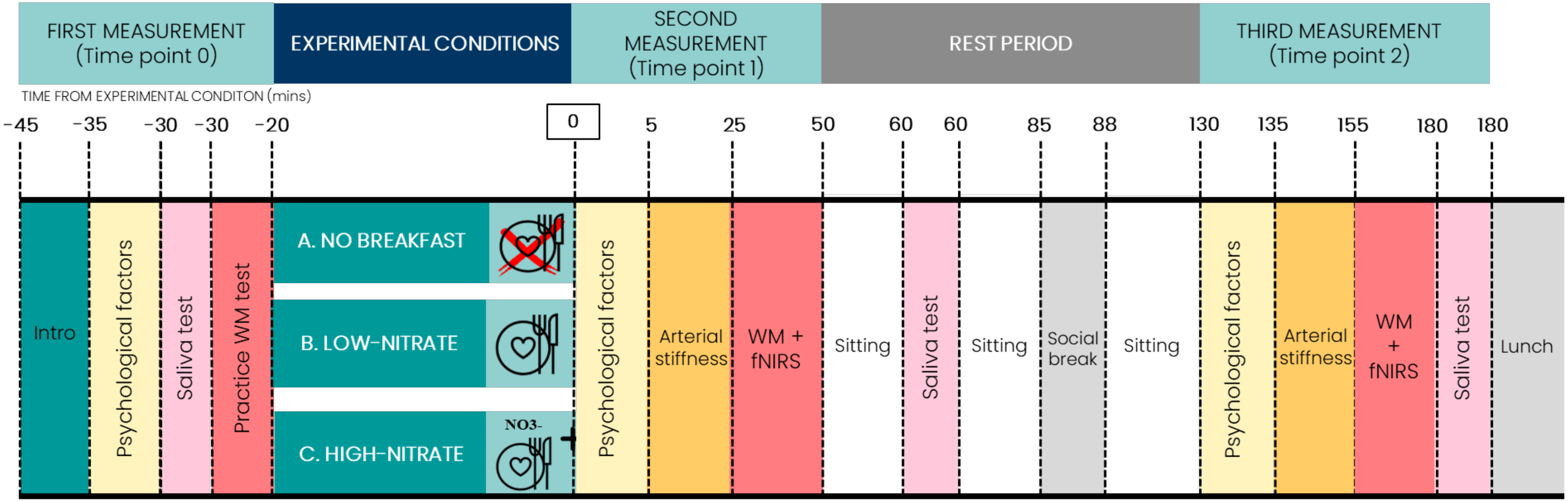

Supplementary Figure 1. Timeline of measurements and experimental conditions. WM = working memory; fNIRS = functional near-infrared spectroscopy.

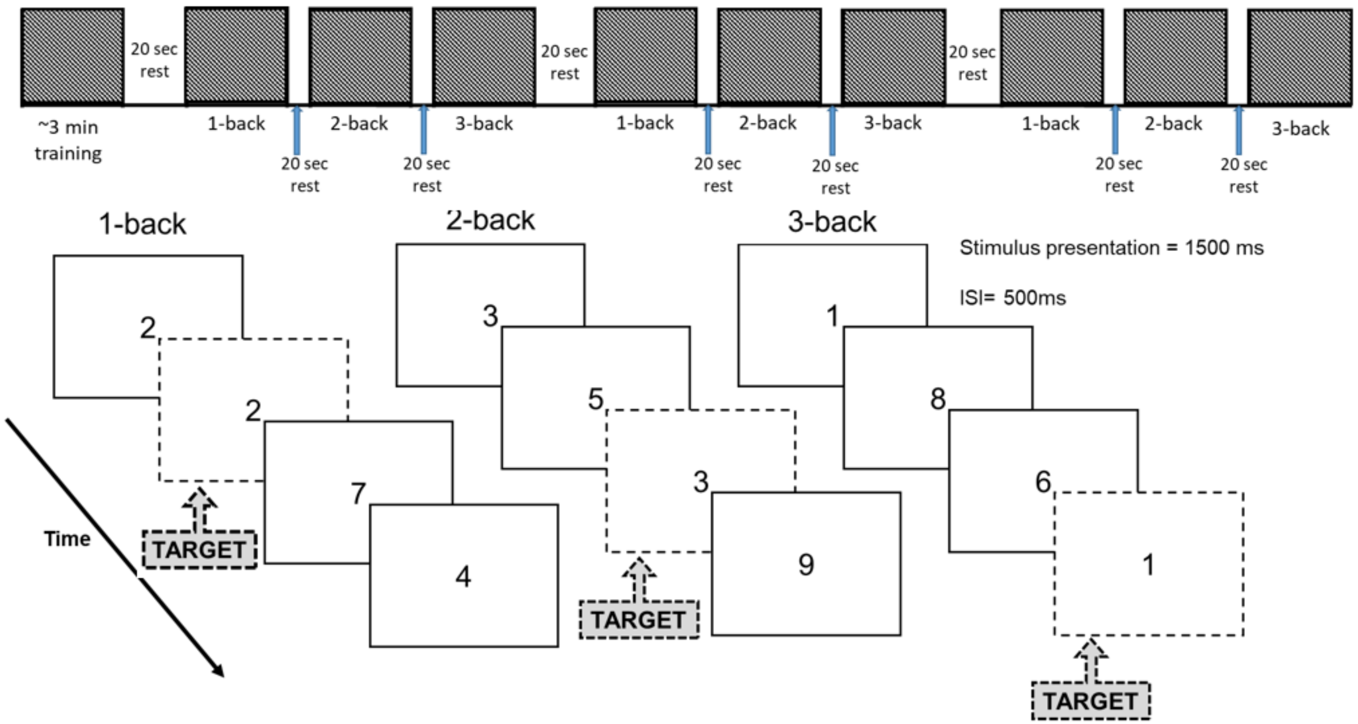

Supplementary Figure 2. Schematic of the working memory tasks to measure cognitive function. Adapted from Regan et al., 2023 (28). ISI = inter-stimulus interval.

Supplementary Figure 3 and Table 1. Functional near-infrared spectroscopy probe design and optode placement according to the 10-20 EEG system using fOLD toolbox*. Channels used for estimating regions of interest. Included in the table are channel, source, and detector coordinating with the figure on the right, and the Montreal Neurological Institute (MNI) coordinates (X,Y,Z), anatomical landmarks, and specificity. In the figure: SD= short distance. Black circles = sources; Gray circles = detectors. Channel number is indicated in blue.

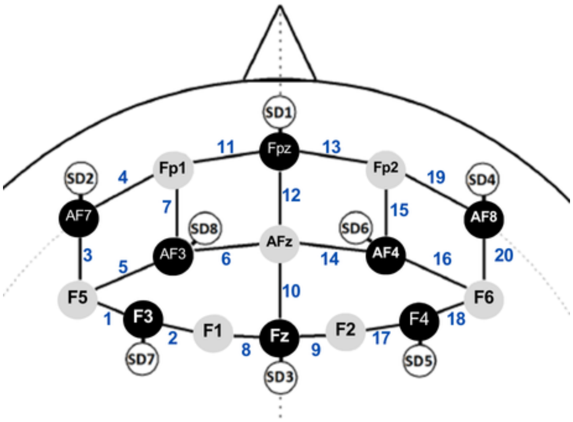

Channel	Source	Detector	Brodmann's area	Anatomical landmark	MNI coordinate (mm)			Specificity	Distance
					X	Y	Z	%	mm
**LEFT HEMISPHERE**									
1	F3	F5	45	Pars triangularis Broca's areas	-46	39	26	72.6	29
3	AF7	F5	45	Pars triangularis Broca's areas	-47	46	6	48.8	33
5	AF3	F5	46	DLPFC	-39	50	17	49.3	44
4	AF7	Fp1	11	Orbitofrontal area	-33	59	-2	32.7	30
6	AF3	AFz	10	Frontopolar area	-12	62	23	75.8	36
7	AF3	Fp1	10	Frontopolar area	-24	63	9	69.6	27
11	Fpz	Fp1	10	Frontopolar area	-12	67	0	54.5	30
2	F3	F1	9	DLPFC	-31	39	41	66.6	29
8	Fz	F1	9	DLPFC	-9	41	50	63.2	29
**MIDDLE**									
10	Fz	AFz	9	DLPFC	2	50	39	61.8	40
12	Fpz	AFz	10	Frontopolar area	1	64	14	87.5	40
**RIGHT HEMISPHERE**									
9	Fz	F2	9	DLPFC	10	41	50	68.9	29
17	F4	F2	9	DLPFC	30	40	41	68.4	30
13	Fpz	Fp2	10	Frontopolar area	13	67	0	54.5	31
14	AF4	AFz	10	Frontopolar area	13	61	24	72.5	36
15	AF4	Fp2	10	Frontopolar area	25	63	9	68.8	28
19	AF8	Fp2	10	Frontopolar area	34	59	-2	31.1	30
18	F4	F6	45	Pars triangularis Broca's areas	46	38	24	70.7	30
20	AF8	F6	45	Pars triangularis Broca's areas	48	46	5	43.9	33
16	AF4	F6	46	DLPFC	40	50	16	47.4	45

DLPFC= Dorsolateral prefrontal cortex; MNI = Montreal Neurological Institute. *Zimeo Morais GA et al. fNIRS Optodes' Location Decider (fOLD): a toolbox for probe arrangement guided by brain regions-of-interest, Scientific reports. 2018;8(1):3341.

Supplementary Table 2. Number of participants analysed per condition for different measurement outcomes.

Experimental condition	Working memory and fNIRS outcomes	Other outcome variables
No Breakfast	57	60
Low nitrate (regular breakfast)	57	57 (except PANAS N=56)
High-nitrate breakfast	55	55

fNIRS: functional near-infrared spectroscopy.

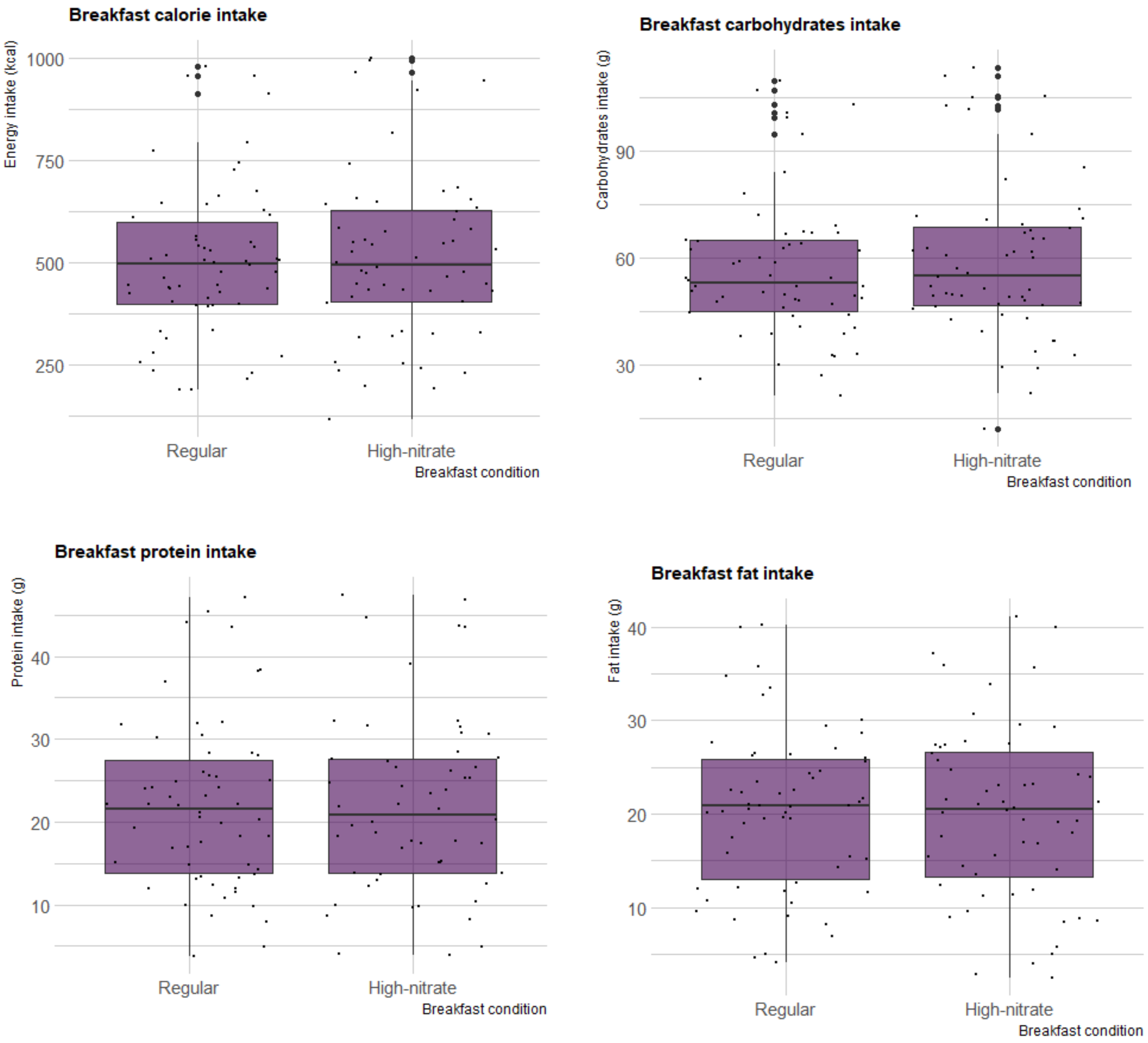

Supplementary Figure 4. Boxplots of individual and median level intake of energy (kilocalories), protein (grams), fat (grams), and carbohydrates (grams) for the regular and high-nitrate breakfast conditions.

Supplementary Table 3. Average with confidence interval, and beta change for accuracy and reaction time in the working memory tests.

	**ACCURACY**			**REACTION TIME**		
	No breakfast			No breakfast		
	Average (95% CI)		βΔ (95% CI)	Average (95% CI)		βΔ (95% CI)
	T1	T2	βT2-T1	T1	T2	βT2-T1
1back	53.5(51.5-55.4)	54.9(52.9-56.8)	1.4(-0.3-3.1)	645.2(670.3-620.0)	605.6(630.7-580.4)	**-39.6(-54.8--24.4)****
2back	51.3(49.1-53.5)	52.4(50.2-54.6)	**1.1(0.1-2.1)***	714.2(744.0-684.4)	661.4(691.2-631.6)	**-52.8(-76.3--29.3)****
3back	47.3(44.8-49.8)	48.9(46.4-51.4)	**1.6(0.4-2.9)***	733.5(766.4-700.6)	702.0(734.9-669.1)	**-31.5(-52.5--10.4)***
	Regular breakfast			Regular breakfast		
	T1	T2	βT2-T1	T1	T2	βT2-T1
1back	53.5(51.9-55.1)	54.1(52.5-55.7)	0.6(-0.5-1.7)	643.3(667.4-619.2)	592.3(616.4-568.2)	**-51.0(-68.6--33.3)****
2back	51.5(49.4-53.5)	51.9(49.9-54.0)	0.5(-0.9-1.8)	708.1(742.9-673.2)	662.8(697.6-628.0)	**-45.3(-65.1--25.4)****
3back	47.5(45.2-49.7)	48.1(45.9-50.4)	0.6(-0.7-2.0)	740.7(776.4-705.1)	692.7(728.4-657.1)	**-48.0(-66.3--29.6)****
	High-nitrate breakfast			High-nitrate breakfast		
	T1	T2	βT2-T1	T1	T2	βT2-T1
1back	53.9(52.3-55.5)	55.9(54.3-57.5)	**2.1(0.8-3.3)***	620.3(645.4-595.3)	586.0(611.1-560.9)	**-34.3(-48.5--20.2)****
2back	51.3(49.1-53.5)	53.2(51.0-55.3)	**1.9(0.7-3.1)***	682.4(714.8-650.0)	654.6(687.0-622.2)	**-27.8(-44.5--11.1)****
3back	47.1(44.5-49.7)	49.4(46.9-52.0)	**2.3(1.1-3.6)****	718.6(752.6-684.5)	677.6(711.7-643.5)	**-41.0(-60.9--21.1)****

*P≤0.05; **P≤0.001; CI: confidence interval.

Supplementary Table 4. Change from time point 1 to time point 2 of oxygenated- and deoxygenated-haemoglobin during the 2- and 3-back tests compared to the 1-back in the different conditions.

		Left prefrontal cortex		Right prefrontal cortex	
		βΔOxy-Hb T2-T1	βΔDeoxy-Hb T2-T1	βΔOxy-Hb T2-T1	βΔDeoxy-Hb T2-T1
No breakfast	2-back	-0.054(-0.074--0.034)**	0.028(0.017-0.040)**	-0.018(-0.035--0.001)	0.013(0.002-0.024)*
	3-back	-0.008(-0.027-0.012)	0.004(-0.007-0.016)	0.007(-0.010-0.023)	0.011(0.000-0.021)
Regular breakfast	2-back	-0.054(-0.073--0.036)**	0.017(0.005-0.028)**	-0.044(-0.060--0.029)**	0.029(0.019-0.039)**
	3-back	-0.063(-0.081--0.044)**	0.026(0.014-0.037)**	-0.050(-0.065--0.034)**	0.035(0.024-0.046)**
High-nitrate breakfast	2-back	0.014(-0.007-0.035)	-0.012(-0.024--0.0002)	0.002(-0.017-0.020)	-0.001(-0.012-0.010)
	3-back	0.017(-0.005-0.038)	-0.017(-0.028--0.005)*	0.050(0.031-0.069)**	-0.025(-0.036--0.013)**

*q adjusted for multiple comparisons ≤0.05; **q ≤0.001;

Supplementary Table 5. Between condition differences in oxygenated- and deoxygenated-haemoglobin, from pre to post, compared to the 1-back.

		Left prefrontal cortex				Right prefrontal cortex			
**BREAKFAST**		**βΔOxy-Hb**	**d**	**βΔDeoxy-Hb**	**d**	**βΔOxy-Hb**	**d**	**βΔDeoxy-Hb**	**d**
Regular - No	2-back	-0.001 (-0.027, 0.026)	-0.002	-0.012 (-0.028, 0.004)	-0.09	-0.026 (-0.049,-0,003)	-0.14	0.016 (0.001, 0.031)	0.13
	3-back	-0.055 (-0.083 ,-0.028)	-0.25	0.021 (0.005, 0.038)	0.16	-0.056 (-0.079, -0.034)	-0.31	0.024 (0.009, 0.039)	0.20
High-nitrate - No	2-back	0.068 (0.039 ,0.097)	0.29	-0.040 (-0.056, -0.024)	-0.31	0.020 (-0.005, 0.045)	0.10	-0.014 (-0.030, 0.001)	-0.11
	3-back	0.024 (-0.005, 0.053)	0.10	-0.021 (-0.037, -0.004)	-0.16	0.043 (0.018, 0.068)	0.21	-0.036 (-0.051, -0.020)	-0.28
High-nitrate - Regular	2-back	0.068 (0.041 ,0.096)	0.31	-0.029 (-0.045, -0.012)	-0.22	0.046 (0.022 ,0.070)	0.24	-0.030 (-0.045, -0.015)	-0.24
	3-back	0.080 (0.051, 0.108)	0.35	-0.042 (-0.059, -0.026)	-0.32	0.100 (0.075, 0.124)	0.50	-0.060 (-0.076, -0.044)	-0.47

*q adjusted for multiple comparisons ≤0.05; **q ≤0.001; d = Cohen's d

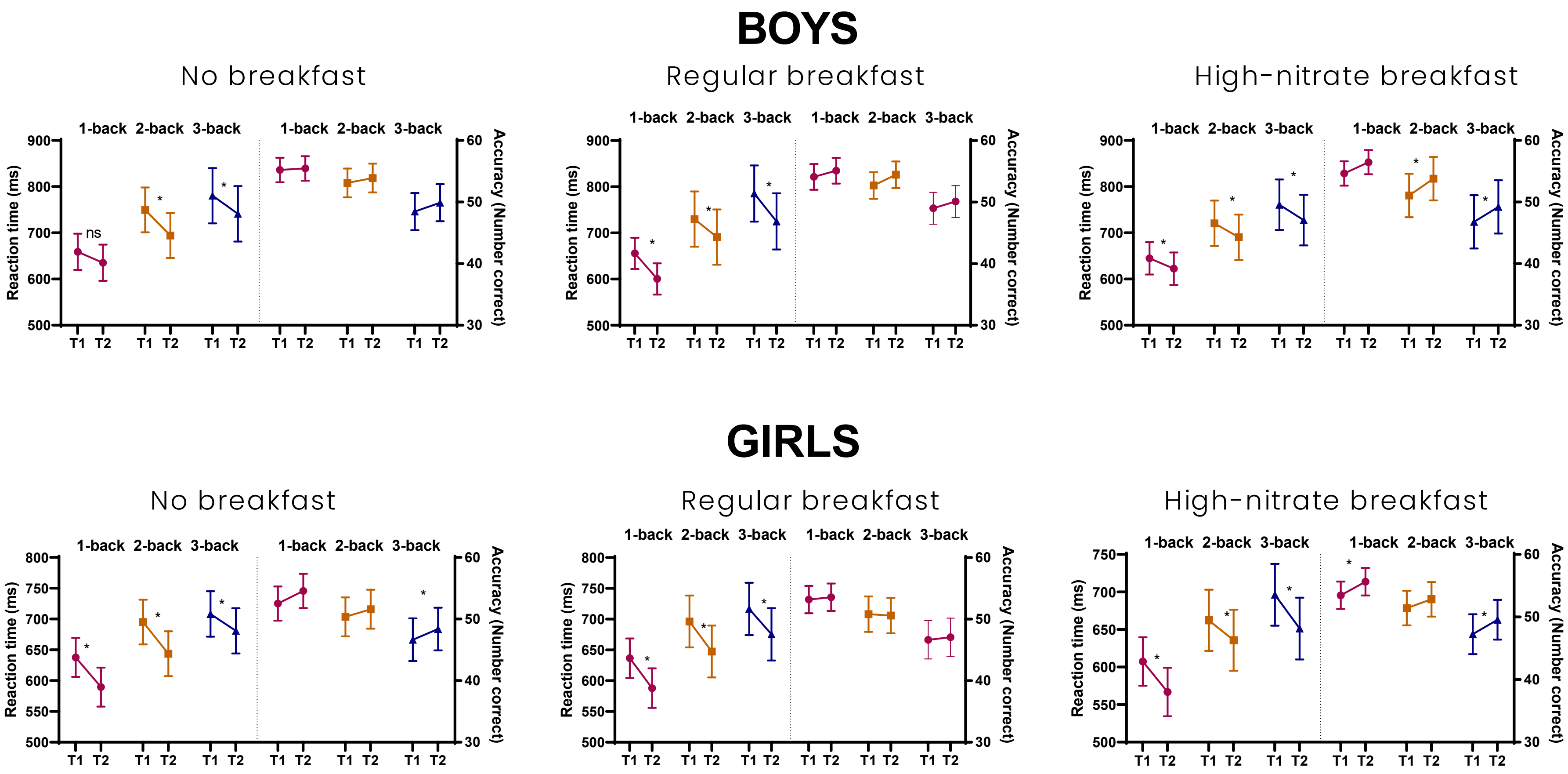

Supplementary Figure 5. Within condition changes in working memory stratified by boys and girls.

Changes in reaction time and accuracy in the different breakfast conditions (N=60) for the 1-, 2-, and 3-back tests from time point 1 (T1) to time point 2 (T2). **P*<0.05; ***P*<0.01; ns = *P*≥0.05.

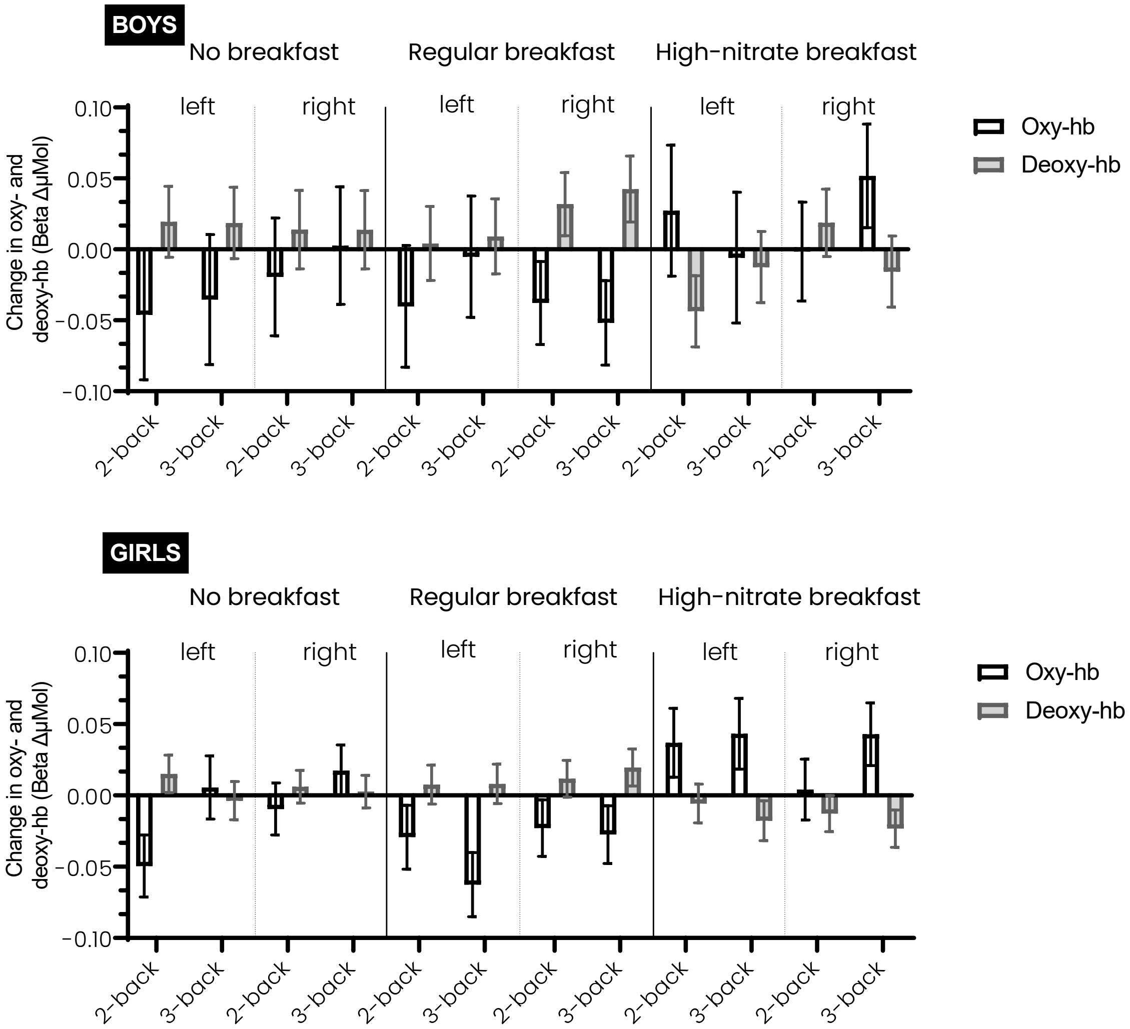

Supplementary Figure 6. Within condition changes in oxygenated and deoxygenated haemoglobin concentrations stratified by boys and girls.

Change from time point 1 to time point 2 of oxygenated (Oxy-hb) and deoxygenated (Deoxy-hb) haemoglobin in the 2-back and 3-back tests relative to the 1-back, stratified by left and right prefrontal cortex hemisphere (N=60).

Supplementary Table 6. Within condition changes (T2-T1) of oxygenated- and de-oxygenated haemoglobin, comparing 3-back and 2-back tests to the 1-back, by region of interest.

**BA, Anatomical landmark, channel**		**No breakfast**						**Regular Breakfast**						**High-nitrate breakfast**					
		**ΔOxy-Hb**			**ΔDeoxy-Hb**			**ΔOxy-Hb**			**ΔDeoxy-Hb**			**ΔOxy-Hb**			**ΔDeoxy-Hb**		
		β	95%CI		β	95%CI		β	95%CI		β	95%CI		β	95%CI		β	95%CI	
9-DLPFC, left, ch 2,8	3-back	**-0.115**	-0.172	-0.057	0.015	-0.015	0.046	-0.051	-0.100	-0.001	0.028	0.004	0.053	-0.037	-0.101	0.027	-0.011	-0.040	0.019
	2-back	**-0.108**	-0.163	-0.054	**0.055**	0.025	0.085	-0.025	-0.076	0.026	0.022	-0.003	0.046	0.023	-0.042	0.087	-0.021	-0.051	0.009
46-DLPFC, Left, ch 5	3-back	-0.021	-0.080	0.038	-0.003	-0.028	0.022	-0.071	-0.139	-0.004	0.027	-0.004	0.058	-0.068	-0.128	-0.009	-0.003	-0.028	0.022
	2-back	**-0.105**	-0.164	-0.047	**0.033**	0.009	0.057	0.050	-0.017	0.116	-0.013	-0.044	0.017	**-0.085**	-0.142	-0.028	-0.005	-0.029	0.019
45-Pars triangularis Broca's area, left, ch 1,3	3-back	0.019	-0.040	0.079	-0.016	-0.040	0.008	**-0.066**	-0.108	-0.024	**0.030**	0.012	0.048	**0.098**	0.044	0.152	-0.012	-0.034	0.009
	2-back	-0.003	-0.061	0.056	-0.016	-0.039	0.007	**-0.123**	-0.164	-0.081	**0.039**	0.021	0.057	**0.101**	0.050	0.152	-0.007	-0.028	0.013
11-Orbitofrontal area, left, ch 4	3-back	**0.034**	0.013	0.055	-0.013	-0.033	0.008	0.017	-0.005	0.039	-0.024	-0.045	-0.004	0.012	-0.015	0.039	-0.009	-0.040	0.021
	2-back	-0.008	-0.029	0.013	-0.001	-0.022	0.020	**0.047**	0.025	0.069	**-0.040**	-0.060	-0.019	**0.067**	0.042	0.093	-0.031	-0.061	-0.002
10-Frontopolar area, left, ch 6,7,11	3-back	0.020	-0.011	0.050	0.003	-0.021	0.027	**-0.087**	-0.122	-0.051	0.027	0.001	0.053	0.003	-0.034	0.040	-0.023	-0.048	0.002
	2-back	**-0.041**	-0.072	-0.010	**0.030**	0.007	0.054	**-0.118**	-0.152	-0.083	**0.039**	0.014	0.064	-0.034	-0.070	0.003	-0.015	-0.040	0.010
9-DLPFC, middle, ch 10	3-back	-0.013	-0.026	0.000	0.020	0.000	0.040	**-0.021**	-0.034	-0.007	**0.032**	0.011	0.052	0.000	-0.011	0.012	0.000	-0.019	0.018
	2-back	**-0.043**	-0.056	-0.030	**0.066**	0.046	0.085	-0.002	-0.015	0.011	0.003	-0.017	0.024	-0.006	-0.017	0.006	0.009	-0.008	0.026
10-Frontopolar, middle, ch 12	3-back	0.048	0.001	0.096	**0.034**	0.005	0.063	**-0.123**	-0.175	-0.071	**0.050**	0.019	0.080	**0.111**	0.049	0.172	**-0.055**	-0.088	-0.022
	2-back	**-0.094**	-0.141	-0.047	**0.047**	0.018	0.075	-0.046	-0.096	0.005	-0.005	-0.034	0.024	0.031	-0.029	0.090	0.001	-0.028	0.029
9-DLPFC, right, ch 9,17	3-back	-0.007	-0.048	0.034	0.003	-0.025	0.031	-0.025	-0.058	0.008	0.016	-0.010	0.041	**0.063**	0.021	0.106	-0.016	-0.047	0.014
	2-back	**-0.065**	-0.108	-0.022	0.007	-0.020	0.035	**-0.053**	-0.085	-0.022	0.001	-0.023	0.026	**-0.082**	-0.124	-0.041	-0.002	-0.032	0.028
10-Frontopolar area, right, ch 13,14,15,19	3-back	-0.007	-0.042	0.029	0.008	-0.015	0.031	**-0.068**	-0.108	-0.027	**0.075**	0.049	0.100	-0.014	-0.049	0.021	-0.020	-0.047	0.008
	2-back	-0.012	-0.049	0.024	0.029	0.005	0.053	-0.042	-0.082	-0.001	0.025	-0.001	0.052	0.020	-0.014	0.055	-0.016	-0.043	0.011
45-Pars triangularis Broca's area, right, ch 2, 18	3-back	0.043	-0.002	0.088	0.004	-0.020	0.027	-0.016	-0.057	0.026	0.012	-0.010	0.034	0.007	-0.049	0.062	0.001	-0.026	0.029
	2-back	0.057	0.010	0.104	0.006	-0.018	0.030	0.040	-0.002	0.081	0.002	-0.019	0.024	-0.072	-0.124	-0.020	0.033	0.006	0.060
46-DLPFC, right, ch 16	3-back	-0.006	-0.015	0.002	0.010	-0.003	0.022	**-0.036**	-0.043	-0.028	**0.054**	0.042	0.066	0.009	-0.001	0.018	-0.013	-0.028	0.001
	2-back	0.005	-0.003	0.013	-0.008	-0.020	0.005	**-0.028**	-0.035	-0.020	**0.042**	0.031	0.054	-0.002	-0.011	0.007	0.003	-0.011	0.017

Bold indicates q-value for multiple comparison <0.05; BA = Brodmann’s area; Oxy-Hb = oxygenated haemoglobin; Deoxy-Hb = deoxygenated haemoglobin; DLPFC = dorsolateral prefrontal cortex; Ch = channel; CI=confidence interval.

Supplementary Table 7. Between condition differences in oxygenated- and deoxygenated-haemoglobin, from pre to post-test, compared to the 1-back.

			**2-back**						**3-back**					
			**Oxy-Hb**			**Deoxy-Hb**			**Oxy-Hb**			**Deoxy-Hb**		
**Channel**	**BA-Anatomical Landmark**	**Breakfast contrast**	**βΔ**	**95%CI**		**βΔ**	**95%CI**		**βΔ**	**95%CI**		**βΔ**	**95%CI**	
2,8	9-DLPFC, left	Regular - No	0.084	0.009	0.159	-0.033	-0.072	0.006	0.064	-0.011	0.140	0.013	-0.026	0.052
		High-nitrate - No	**0.131**	0.047	0.216	**-0.076**	-0.118	-0.034	0.078	-0.008	0.164	-0.026	-0.068	0.016
		High-nitrate - Regular	0.047	-0.035	0.129	-0.043	-0.082	-0.004	0.014	-0.068	0.095	-0.039	-0.078	0.000
5	46-DLPFC, Left	Regular - No	**0.155**	0.067	0.243	-0.046	-0.085	-0.007	-0.050	-0.140	0.040	0.030	-0.010	0.070
		High-nitrate - No	0.020	-0.062	0.101	-0.038	-0.071	-0.004	-0.047	-0.132	0.037	0.000	-0.035	0.035
		High-nitrate - Regular	**-0.135**	-0.222	-0.048	0.009	-0.030	0.047	0.003	-0.087	0.093	-0.030	-0.070	0.010
1,3	45-Pars triangularis Broca's area, left	Regular - No	**-0.120**	-0.192	-0.049	**0.055**	0.026	0.084	**-0.085**	-0.158	-0.013	0.046	0.016	0.076
		High-nitrate - No	**0.104**	0.026	0.181	0.009	-0.022	0.039	0.078	-0.002	0.159	0.004	-0.028	0.036
		High-nitrate - Regular	**0.224**	0.158	0.290	**-0.046**	-0.073	-0.019	**0.164**	0.095	0.232	**-0.042**	-0.070	-0.014
4	11-Orbitofrontal area, left	Regular - No	**0.055**	0.025	0.085	**-0.039**	-0.068	-0.010	-0.017	-0.048	0.014	-0.012	-0.041	0.018
		High-nitrate - No	**0.075**	0.042	0.108	-0.030	-0.067	0.006	-0.022	-0.056	0.013	0.004	-0.033	0.040
		High-nitrate - Regular	0.020	-0.013	0.054	0.008	-0.028	0.044	-0.005	-0.040	0.031	0.015	-0.022	0.052
6,7,11	10-Frontopolar area, left	Regular - No	**-0.077**	-0.124	-0.031	0.009	-0.025	0.043	**-0.107**	-0.154	-0.060	0.024	-0.011	0.059
		High-nitrate - No	0.007	-0.041	0.055	**-0.046**	-0.080	-0.011	-0.017	-0.065	0.031	-0.026	-0.060	0.009
		High-nitrate - Regular	**0.084**	0.034	0.135	**-0.055**	-0.090	-0.019	**0.089**	0.038	0.141	**-0.050**	-0.086	-0.014
10	9-DLPFC, middle	Regular - No	**0.041**	0.022	0.059	**-0.063**	-0.091	-0.034	-0.008	-0.026	0.011	0.012	-0.017	0.040
		High-nitrate - No	**0.037**	0.020	0.054	**-0.057**	-0.083	-0.031	0.013	-0.004	0.031	-0.020	-0.047	0.007
		High-nitrate - Regular	-0.004	-0.021	0.014	0.006	-0.021	0.033	**0.021**	0.003	0.039	**-0.032**	-0.060	-0.004
12	10-Frontopolar, middle	Regular - No	0.048	-0.021	0.117	**-0.052**	-0.093	-0.011	**-0.171**	-0.242	-0.101	0.016	-0.026	0.058
		High-nitrate - No	**0.124**	0.049	0.200	**-0.046**	-0.087	-0.005	0.062	-0.016	0.140	**-0.089**	-0.133	-0.045
		High-nitrate - Regular	0.076	-0.001	0.154	0.006	-0.035	0.047	**0.234**	0.153	0.314	**-0.105**	-0.150	-0.060
9,17	9-DLPFC, right	Regular - No	0.012	-0.042	0.065	-0.006	-0.043	0.031	-0.018	-0.070	0.035	0.012	-0.026	0.050
		High-nitrate - No	-0.018	-0.077	0.042	-0.010	-0.050	0.031	0.070	0.011	0.130	-0.020	-0.061	0.022
		High-nitrate - Regular	-0.029	-0.081	0.023	-0.003	-0.042	0.035	**0.088**	0.035	0.142	-0.032	-0.072	0.008
13,14,15,19	10-Frontopolar area, right	Regular - No	**-0.082**	-0.117	-0.048	**0.064**	0.039	0.090	**-0.065**	-0.100	-0.030	**0.039**	0.013	0.064
		High-nitrate - No	**0.090**	0.054	0.126	-0.025	-0.051	0.001	**0.079**	0.043	0.115	**-0.054**	-0.080	-0.028
		High-nitrate - Regular	**0.172**	0.135	0.209	**-0.090**	-0.116	-0.063	**0.144**	0.106	0.182	**-0.092**	-0.119	-0.065

18,2	45-Pars triangularis Broca's area, right	Regular - No	-0.018	-0.080	0.045	-0.003	-0.036	0.029	-0.059	-0.120	0.003	0.008	-0.024	0.040
		High-nitrate - No	**-0.130**	-0.200	-0.059	0.028	-0.009	0.064	-0.036	-0.108	0.036	-0.002	-0.039	0.034
		High-nitrate - Regular	-0.112	-0.178	-0.046	0.031	-0.004	0.065	0.022	-0.047	0.092	-0.011	-0.046	0.025
16	46-DLPFC, right	Regular - No	**-0.033**	-0.044	-0.022	**0.050**	0.033	0.067	**-0.029**	-0.040	-0.018	**0.044**	0.027	0.062
		High-nitrate - No	-0.007	-0.019	0.005	0.011	-0.008	0.029	**0.015**	0.003	0.028	**-0.023**	-0.042	-0.004
		High-nitrate - Regular	**0.026**	0.014	0.038	**-0.040**	-0.058	-0.021	**0.044**	0.032	0.057	**-0.068**	-0.087	-0.049

Bold indicates q-value for multiple comparison <0.05; BA = Brodmann's area; Oxy-Hb = oxygenated haemoglobin; Deoxy-Hb = deoxygenated haemoglobin; DLPFC = dorsolateral prefrontal cortex; CI=confidence interval.

Supplementary Table 8. Averages and 95% confidence intervals for psychological and peripheral vascular measures at different time points during the breakfast conditions.

	No breakfast			Regular breakfast			High-nitrate breakfast		
	Avg.	Lower 95%CI	Upper 95%CI	Avg.	Lower 95%CI	Upper 95%CI	Avg.	Lower 95%CI	Upper 95%CI
Time					**SLEEPINESS**				
**0**	5.85	5.45	6.25	5.54	5.13	5.95	5.57	5.18	5.96
**1**	5.38	4.98	5.78	4.69	4.28	5.10	4.55	4.15	4.95
**2**	4.77	4.37	5.17	4.43	4.02	4.84	4.24	3.84	4.64
					**ALERTNESS**				
**0**	4.17	3.71	4.62	4.53	4.04	5.02	4.67	4.22	5.11
**1**	4.79	4.33	5.24	5.64	5.16	6.13	5.68	5.24	6.13
**2**	5.42	4.96	5.88	5.71	5.22	6.19	5.90	5.46	6.34
					**POSITIVE MOOD**				
**0**	20.35	18.75	21.95	21.46	19.65	23.27	21.80	20.14	23.47
**1**	20.03	18.44	21.63	22.37	20.56	24.18	21.41	19.73	23.08
**2**	19.95	18.35	21.55	21.22	19.40	23.04	21.88	20.21	23.55
					**NEGATIVE MOOD**				
**0**	13.00	12.17	13.82	13.08	12.00	14.15	13.32	12.54	14.11
**1**	12.71	11.88	13.53	11.82	10.76	12.89	12.39	11.60	13.17
**2**	11.77	10.94	12.59	11.95	10.88	13.02	11.76	10.97	12.55
					**PULSE WAVE VELOCITY (m/s)**				
**1**	4.73	4.59	4.87	4.55	4.38	4.72	4.61	4.48	4.75
**2**	4.64	4.50	4.79	4.55	4.38	4.72	4.53	4.39	4.67
					**AUGMENTATION INDEX (%)**				
**1**	8.85	6.01	11.69	8.71	6.17	11.24	9.20	6.46	11.94
**2**	5.58	2.74	8.43	7.79	5.26	10.33	8.38	5.66	11.11
					**AUGMENTATION INDEX 75 (%)**				
**1**	2.62	-0.43	5.66	4.95	2.21	7.69	5.61	2.61	8.62
**2**	-1.08	-4.13	1.96	3.53	0.80	6.27	3.36	0.38	6.35
					**HEART RATE (beats per minute)**				
**1**	62.13	59.75	64.51	66.97	64.45	69.48	65.26	63.12	67.39
**2**	61.08	58.70	63.46	66.22	63.71	68.74	63.76	61.63	65.90
					**SYSTOLIC BLOOD PRESSURE (mmHg)**				
**1**	108.57	106.62	110.51	109.47	107.66	111.27	109.90	107.97	111.83
**2**	108.27	106.32	110.21	106.95	105.14	108.76	107.15	105.22	109.07
					**DIASTOLIC BLOOD PRESSURE (mmHg)**				
**1**	62.80	61.26	64.34	60.53	59.01	62.06	61.34	59.78	62.89
**2**	60.98	59.45	62.52	58.83	57.31	60.35	59.56	58.01	61.12

Sleepiness measured with the Karolinska Sleepiness Scale, with higher scores indicating more sleepiness. Alertness was measured with a 10-cm visual analogue scale, with a higher score indicating higher alertness Positive and negative mood were derived from the Positive and Negative Affect Scale (PANAS), with higher scores indicating a more positive mood or a more negative mood, respectively. CI: confidence interval.
